# State-of-the-Art Carbon-Nanotubes-Reinforced Copper-Based Composites: The Interface Design of CNTs and Cu Matrix

**DOI:** 10.3390/ijms252312957

**Published:** 2024-12-02

**Authors:** Xiaona Ren, Yue Chang, Changchun Ge

**Affiliations:** 1 School of Materials Science and Engineering, University of Science and Technology Beijing, Beijing 100083, China; ccge@mater.ustb.edu.cn; 2 Institute for Advanced Materials and Technology, University of Science and Technology Beijing, Beijing 100083, China; changyue@ustb.edu.cn

**Keywords:** carbon nanotubes, copper-based composites, chemical bonding, metallurgical bonding, physical binding

## Abstract

Carbon nanotubes (CNTs)-reinforced copper-based composites (CNT/Cu) have been extensively investigated due to their exceptional theoretical electrical, thermal, and mechanical properties. However, the actual performance of these composites has consistently fallen short of theoretical expectations. This discrepancy primarily arises from the inability to achieve direct chemical bonding between copper and carbon nanotubes or to alloy them effectively. Consequently, this leads to interference in electron and phonon transmission at the interface between the two materials, adversely affecting their electrical and thermal conductivity as well as other properties. In recent years, research has increasingly focused on optimizing and regulating the interfacial interactions between carbon nanotubes and the copper matrix to enhance overall performance while also exploring potential applications. This article reviews recent advancements from an interface regulation perspective, summarizing typical interfacial characteristics such as physical interfaces, chemical bonding, and metallurgical bonding along with their respective preparation methods and effects on performance enhancement. Furthermore, a novel microstructural design of CNT/Cu is put forward, where amorphous CNTs (aCNTs) were utilized as the reinforcing phase to form a nanoscale networked composite interface. This not only enables Cu to adhere to the aCNTs’ sidewall but also fills the sidewall within them, with the aim of significantly strengthening the interfacial bonding strength of CNT/Cu and achieving comprehensive improvement of the composite material properties.

## 1. Introduction

Owing to its exceptional electrical and thermal conductivity, workability, and cost-effectiveness, copper (Cu) has been extensively utilized in electronic applications. However, its relatively low hardness and strength have constrained the broader utilization and further development of Cu-based composites, particularly due to its susceptibility to softening and deformation at elevated temperatures [1]. Consequently, copper-based materials have been developed and are widely recognized for their outstanding corrosion resistance, electrical conductivity, thermal conductivity, and ductility. As a result, they play a critical role as essential materials in the power generation and thermal industries.

Since their first discovery in 1991, and especially since around 2000, carbon nanotubes (CNTs) have emerged as a preeminent material, eliciting considerable attention since their advent due to their low density, high rigidity, and extraordinary physicochemical properties [2]. For instance, the distinctive tubular structure of CNTs facilitates the interconnection between the interior and exterior of phase change material (PCM), thereby enhancing its thermal conductivity [3], generates an abnormal anti-Stokes Raman scattering (AASR) phenomenon for detection and analysis [4]. Moreover, CNTs serve as templates for synthesizing nanomaterials [5]. Notably, leveraging these aforementioned properties, the high-aspect-ratio structure and exceptional mechanical characteristics of CNTs make them ideal reinforcing phases and organic fillers that enhance the mechanical, electrical, thermal, or tribological properties of composites [6,7,8,9,10,11]. The integration of CNTs into a Cu matrix can augment the mechanical properties of Cu while conserving its electrical attributes. According to their structure, CNTs are categorized into single-walled carbon nanotubes (SWCNTs), double-walled carbon nanotubes (DWCNTs), multi-walled carbon nanotubes (MWCNTs), and amorphous carbon nanotubes (aCNTs) [12]. Among them, it is widely acknowledged that SWCNTs possess an exceptionally ideal structure and performance, with their conductivity being controllable through chirality regulation. However, surface modification in practical applications cannot be avoided, as it easily compromises their intrinsic structure and impacts their performance. While each layer of MWCNTs exhibits distinct chiralities, determining the conductivity characteristics of MWCNTs remains challenging. Surface modification has minimal influence on the overall structure while ensuring relatively complete retention of performance. Consequently, MWCNTs find predominant utility in practical applications as demonstrated by Table 1, Table 2 and Table 3. Their far-reaching influence encompasses various domains such as physics, chemistry, materials science, and electronics, manifesting vast potential for diverse applications. The remarkable elastic modulus, thermal conductivity, electrical conductivity, and low coefficient of thermal expansion displayed by CNTs facilitate enhancements in hardness as well as thermal and electrical conductivity when combined with metals while maintaining lightweight characteristics [13]. Theoretical findings suggest that SWCNT/Cu composites exhibit 50% of the room-temperature resistivity of Cu [14,15]. Particularly, it has been reported that the incorporation of CNTs can enhance both the electrical conductivity and mechanical properties of low-strength Cu matrices [16,17]. Furthermore, CNT/Cu composites showcase enhanced mechanical strength and electrical conductivity, positioning them as auspicious lightweight electrical conductors for a multiplicity of applications [18,19].

The inclusion of CNTs not only engenders fine-grain strengthening but also fortifies the Cu matrix. The augmentation of composite hardness is invariably concomitant with a reduction in the wear rate; for example, the wear resistance of the CNT/Cu composite is found to be superior to that of the blank control Cu by 4 to 5 times [20]. Conversely, the aggregation of CNTs within copper exerts an adverse impact on the matrix, constituting one of the prevalent issues related to CNT application.

The homogeneous distribution of CNTs within the Cu matrix and robust interfacial adhesion are extensively recognized as pivotal attributes for attaining exceptional properties in CNT/Cu composites. These traits are concurrently manifested in the Cu@CNT/Cu composite, which showcases both admirable strength (272 MPa) and ductility (14.2%) (Table 2) [21]. Additionally, the encapsulation of Cu within CNTs augments the electrical and mechanical attributes of the composite.

The tribological behavior of Cu can be conspicuously enhanced through CNT reinforcement; precisely, HNO_3_-oxidized MWCNTs at a volume fraction of less than 15% have been demonstrated to reduce both the friction coefficient and the wear rate due to the formation of a carbonaceous film at the contact interface, while the self-lubricating property of CNTs mitigates frictional heating [20]. As the concentration of CNTs escalates, the wear mechanism undergoes a transition from plastic deformation to flake formation/spalling.

Accordingly, CNT/Cu composites are prevalently utilized as the detection electrodes for enzyme-free glucose [22,23], carbohydrates [24], and plant virus [25] sensing applications, concurrently functioning as connectors in integrated circuits [26,27] and lightweight electrical wires [18,19,28,29]. Nevertheless, they are more extensively applied in reinforcing copper matrices [30,31,32,33,34,35,36,37,38,39,40] and enhancing their tribological attributes [20,41,42]. Research has manifested that the integration of CNTs into Cu markedly elevates its specific conductivity to 98% of that of pure copper, attributed to the extraordinary elastic modulus, thermal conductivity, electrical conductivity, and low coefficient of thermal expansion exhibited by CNTs. Furthermore, this metal/CNT composite exhibits augmented hardness along with enhanced thermal and electrical conductivity in comparison to their pure metal equivalents [29]. This reinforcing effect contributes not only to superior frictional characteristics but also to specific conductivity. Derived from the conventional CNT-reinforced Cu matrix composites, the laminated CNT/Cu composite—designated as an ultraconductive copper (UCC) composite—has attracted escalating attention [43]. Additionally, the aligned SWCNTs-reinforced Cu demonstrates comparable conductivity (2.3~4.7 × 10^5^ S/cm) to Cu (5.8 × 10^5^ S/cm) while presenting an ampacity (6 × 10^8^ A/cm^2^) up to 100 times higher, signifying the auspicious prospects of CNT/Cu composites in microscale electronics and inverters [44]. The low density and temperature stability of this material make it a promising candidate for replacing copper in the electronics and electrical industries in the future [45].

Presently, investigations on the attributes of CNT/Cu composites mainly center on their electrical and mechanical traits. The as-obtained CNT/Cu exhibits exceptional properties, such as a remarkably high yield strength of 692 MPa [46], a notable tensile strength of 470 MPa, and an impressive electrical conductivity of 98 IACS% [47]. Furthermore, it demonstrates commendable tensile strength (315 MPa), electrical conductivity (94.9 IACS%), and thermal conductivity (416 W/m·K) [48]. Nevertheless, the holistic performance of the obtained CNT/Cu composites lags conspicuously behind the theoretical benchmarks. This disparity is impacted by factors such as the mass, stability, dispersion of CNTs within the Cu matrix, and the quality of interfacial adhesion between the Cu and CNT [15,29,34,49]. Studies have demonstrated a negative correlation between the dissemination of CNTs in the Cu matrix and electrical conductivity [50], intimating that an augmented composite efficacy can be attained via an ordered configuration of CNT arrays within Cu [29,51]. Nonetheless, even when CNTs are systematically arrayed in Cu to enhance thermal conductivity compared to disorderly dispersed composites, the overall functionality might still be undermined due to insufficient interfacial bonding and complications related to particle amalgamation [52].

The restricted application potential of CNT/Cu composite materials emanates from their incapability to constitute alloys and matters concerning wettability and interfacial bonding, which give rise to discontinuities in physicochemical properties [18,53]. For CNTs to augment the metal matrix, several mechanisms are implicated: (a) load transfer from the matrix to CNTs, (b) grain refinement, (c) texture strengthening via CNT pinning, (d) dispersion strengthening by CNTs, (e) solution strengthening by virtue of carbon atoms, and (f) thermal mismatch between CNTs and the matrix [54]. The first two mechanisms predominantly transpire in CNT/Cu composites. Nevertheless, efficacious load transfer between the matrix and CNTs invariably demands a robust bonding between them. Moreover, the precondition for grain refinement is the homogeneous distribution of CNTs within copper.

**Figure 1:**
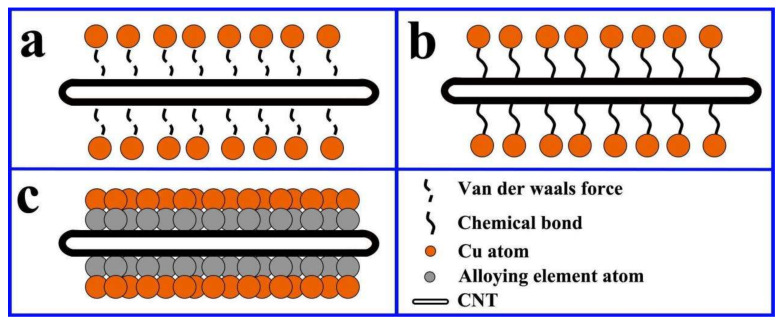
Schematic diagram of the typical interface adhesion: (**a**) physical binding, (**b**) chemical bonding, and (**c**) metallurgical bonding.

**Figure 2:**
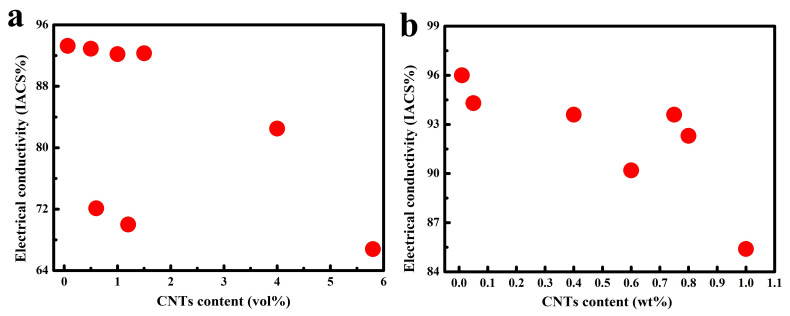
Comparative analysis of electrical conductivity in relation to Table 2, the content of CNTs is measures in vol% (**a**) [64,67,68,69,72,75,77] or wt% (**b**) [21,73,76,78,79,83].

**Figure 3:**
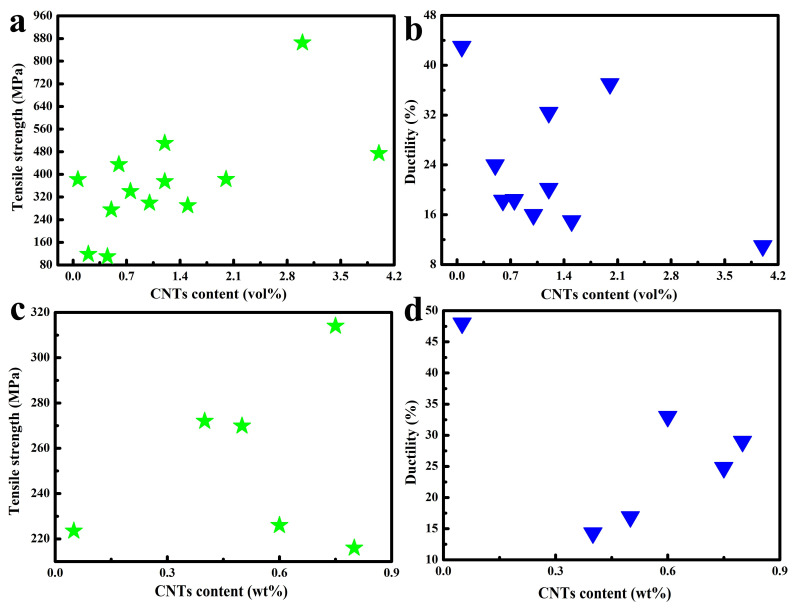
Comparative analysis of tensile strength and ductility in relation to Table 3, the content of CNTs is measures in vol% (**a**) [43,46,64,66,67,68,69,72,74,77,81,82] and (**b**) [64,67,68,69,72,74,77,81,82] or wt% (**c**) [21,71,76,78,79] and (**d**) [21,71,76,78,79].

Moreover, the electrical and thermal conductivity of the composite material are dictated by the interfacial configuration between carbon nanotubes and the copper matrix, where the scattering of phonons and electrons occurs [1,55,56,57,58]. This interfacial construct acts as a hindrance to electron and phonon flux, giving rise to a decreased overall electron–phonon conductance rate; this notwithstanding, CNT/Cu composites are perceived as prospective contenders for electronic materials. As a consequence, modifying the binding modalities and enhancing the interfacial interactions between CNTs and Cu possess considerable potential for concurrently enhancing their mechanical attributes and electrical conductivity [14]. Hence, a crucial facet in the fabrication of CNT/Cu composites resides in attaining effective adhesion between CNTs and Cu. Currently, this is predominantly realized through electrochemical deposition [18,22,23,26,27,30,31], electroless plating [32,33,44,52,59], mechanical mixing techniques such as ball milling or stirring [16,17,19,24,34,35,36,41,50,60,61,62], wet chemistry approaches [37,38,39,63], in situ growth [64,65], and magnetic control sputtering [28] (Table 1). The interface constitutes one of the most pivotal microstructures for composites, especially as carbon and copper do not form any compounds or alloyed architectures. Herein, the interfacial bonding characteristics of carbon-nanotube-reinforced copper matrix composites are comprehensively recapitulated in terms of their research progressions: physical binding, chemical bonding, and metallurgical bonding (Figure 1). According to different bonding methods and preparation processes, the properties of the resulting composites also vary (Figure 2 and Figure 3). This paper focuses on interface composite techniques for CNTs and a Cu matrix, reviewing recent relevant research in order to identify ways to further enhance the properties of CNTs-reinforced Cu matrix composites.

**Table 1:** Fabrication of the CNT/Cu composites.

Method	Precursor	Product Description	Interface Features of Cu and CNTs		Ref.
Brush coating	Cu foils and carboxyl-modified SWCNTs	Cu-CNT-Cu layer by layer	Physical binding	Cu diffusion in CNTs	[43]
Electrospinning-based polymer nanofiber templating and magnetron-sputtering	Cu tapes and carboxyl-modified SWCNTs				[66]
Ball milling, hot pressing, and high-pressure torsion	Electrolytic Cu powder and MWCNTs	CNT/Cu composite			[67]
Co-deposition and SPS	CNTs and CuSO_4_·5H_2_O			Clear and tightly bonded interface	[68]
In situ catalytic growth and SPS	Cu-Al_2_O_3_, C_2_H_4_, H_2_	CNT/Cu-Al_2_O_3_ composite		Tightly bonded interface	[65]
Template, cold pressing, and SPS	CNTs, RGO, CuSO_4_·5H_2_O, and Cu powder	CNTs-RGO/Cu_f_ ^®^Cu		Physical binding between CNTs and Cu	[69]
refined two-step organic–aqueous electrodeposition and hot compression	CNTs, Cu(CH_3_COO)_2_·H_2_O, CuSO_4_·5H_2_O	layered CNT/Cu composite		---	[70]
Electroless deposition	CuSO_4_·5H_2_O and MWCNTs	CNT/Cu thin film	Chemical bonding	Carboxyl or tiol	[14]
Wet-chemical, ball milling, and SPS	Cu(CH_3_COO)_2_·H_2_O and graphitized CNTs	CNT/Cu composite		Cu_2_O	[21]
Wet mixing, thermal reduction, and SPS	Modified MWCNTs and Cu powders				[71]
Molecular-level mixing, ball milling, and thermal reduction	Cross-linked modified CNTs and Cu (CH_3_COO)_2_·H_2_O				[72]
Vacuum mixing and SPS	Modified MWCNTs, fine and coarse Cu powders				[73]
Electroless deposition, slurry dispersion, ball milling, cold isostatic pressing, vacuum sintering, and hot extrusion	Modified MWCNTs, Cu and TiB_2_ powders	Cu matrix composites reinforced with MWCNTs and TiB_2_ microparticles			[74,75]
Spraying pyrolysis, low-energy ball milling, and SPS	Modified and W-coated MWCNTs, Cu powders	CNT-W/Cu composite			[76]
Electroless deposition, ball milling, internal oxidation, and hot extrusion	CNTs, gas-atomized Cu-0.8Al powders, and Cu_2_O powders	Cu-Al_2_O_3_-CNT composite		Cu_x_O_y_	[77]
Electrodeposition, ball milling, and SPS	AgNO_3_, MWCNTs, and electrolytic Cu powder	Nanocomposites of Ag-nanoparticle-coated CNTs are uniformly distributed in Cu	Metallurgical bonding	Ag improved surface wettability	[78]
Ball milling and SPS	Cu powder, 4 at%Ni-1 at%Y catalyzed SWCNTs	---		Ni-decorated SWCNTs	[79]
Ball milling, vacuum hot-pressed sintering, and hot rolling	CNTs, Cr_2_(SO_4_)_3_ and Cu powder	CNT/Cu composite		Cr_3_C_2_ and Cr_23_C_6_	[80]
Catalyzed CVD and SPS	CuCr alloy, Cu powder, H_2_, and C_2_H_4_		Chemical and metallurgical bonding	Cu_2_O and Cr_3_C_2_	[64]
Chemical unzipping, ball milling, and SPS	CuCr alloy powder, CNTs, H_2_SO_4_, and KMnO_4_	CNT/CuCr composite		Cr_7_C_3_ and Cr_23_C_6_	[81]
Chemical modification and plating, ball milling, and fast hot-pressing sintering	CNTs, Cu powder	CNT/Cu composite		Cu_2_O and Sn and Ag diffused to Cu	[82]

**Table 2:** Electrical properties of CNT/Cu with different interface features.

Interface Features	Strengthening Phase	Modified Group	Content	Electrical Conductivity (IACS%)	Ref.
Physical binding	MWCNTs	---	4 vol%	82.5 ± 1.1	[67]
	CNTs		1.5 vol%	92.3	[68]
			---	81.4	[70]
	CNTs and Al_2_O_3_		---	83.2	[65]
	CNTs and RGO		0.064 vol%	93.26	[69]
Chemical bonding	modified SWCNTs	-COOH	~0.45–0.5 vol%	---	[43]
			~0.2 vol%	---	[66]
			0.01 wt%	96	[83]
	Modified MWCNTs	-OH	~16 wt%, ~45 vol%	0.345	[14]
		-SH		1.41	
		-COOH	0.4 wt.%	93.6	[21]
		Oxygen-containing groups	0.8 wt%	92.3	[76]
			1 wt%	85.4 ± 0.6	[73]
		Diazotizing and oxygen-containing groups	1 vol%	92.2 ± 0.4	[72]
	CNTs and Al_2_O_3_	---	0.6 vol% CNTs and 3.5 vol% Al_2_O_3_	72.1 ± 0.8	[77]
	CNTs and TiB_2_	Oxygen-containing groups	5.8 vol% (*x*CNTs + *y*TiB_2_), *x* = 0, 1.4, 2.8, 4.2, 5.8, *x* + *y* = 5.8	58.9 (*x* = 0), 56.9 (*x* = 1.4), 56.6 (*x* = 2.8), 59.2 (*x* = 4.2), 66.8 (*x* = 5.8)	[75]
Metallurgical bonding	Ni-decorated SWCNTs	---	0.05 wt%	94.3 ± 0.9	[79]
	MWCNTs	Ag nanoparticles	0.75 wt% (CNT-Ag composite powder)	93.6	[78]
Multiple interface bonding		Cr_3_C_2_ and Cu_2_O	0.5 vol%	92.9	[64]

**Table 3:** Mechanical properties of CNT/Cu with different interface features.

Interface Features	Strengthening Phase	Content	Hardness (HV)	Tensile Strength (MPa)	Ductility (%)	Ref.
Physical binding	MWCNTs	4 vol%	---	474.3 ± 12.7	11.0 ± 0.5	[67]
	CNTs	1.5 vol%	---	290	15	[68]
		---	---	392	---	[70]
	CNTs and Al_2_O_3_		123.3	280	---	[65]
	CNTs and RGO	0.064 vol%	135.7~141.79 at the pure Cu region, 154.23~161.84 at the skeleton region	382 ± 2	43 ± 0.7	[69]
Chemical bonding	-COOH-modified SWCNTs	~0.45–0.5 vol%	---	~110	---	[43]
		~0.2 vol%	---	~118	---	[66]
		0.01 wt%	61	---	---	[83]
	HNO_3_-oxidized MWCNTs	15 vol%	126	---	---	[20]
	-COOH-modified MWCNTs	0.4 wt.%	---	272	14.3	[21]
	-OH- and -COOH-modified MWCNTs	0.5 wt%	---	269.9	16.9	[71]
	-OH- and/or -COOH-modified MWCNTs	0.6 wt%	---	226	33	[76]
		1 wt%	113 ± 4	---	---	[73]
		3 vol%	---	865		[46]
	-OH- and/or -COOH-modified and diazotized MWCNTs	1 vol%	---	299 ± 8	16 ± 3	[72]
	H_2_SO_4_ (98%)- and HNO_3_ (68%)-oxidized MWCNTs and TiB_2_	1.2 vol% MWCNTs and 4.8 vol% TiB_2_	---	375 ± 5	32.4 ± 2.3	[74]
	CNTs and Al_2_O_3_	1.2 vol% CNTs and 3.5 vol% Al_2_O_3_	---	510 ± 6	20.2	[77]
Metallurgical bonding	Ag-coated MWCNTs	0.75 wt%	85.25	314	24.8	[78]
	Ni-decorated SWCNTs	0.05 wt%	79.1 ± 2.6	223.5 ± 6.5	48 ± 2.5	[79]
	Cr_2_O_3_-decorated CNTs	0.75 vol%	117.3 ± 5.2	---	---	[80]
Multiple interface bonding	CNTs prepared via CuCr-alloy-catalyzed CVD	0.5 vol%	102.5	275	24	[64]
	Partially unzipped CNTs	2 vol%	---	382.9	37.02	[81]
	-OH- and/or -COOH-modified CNTs	0.75 vol%	---	340	18.4	[82]

## 2. Physical Binding Interface

**Figure 4:**
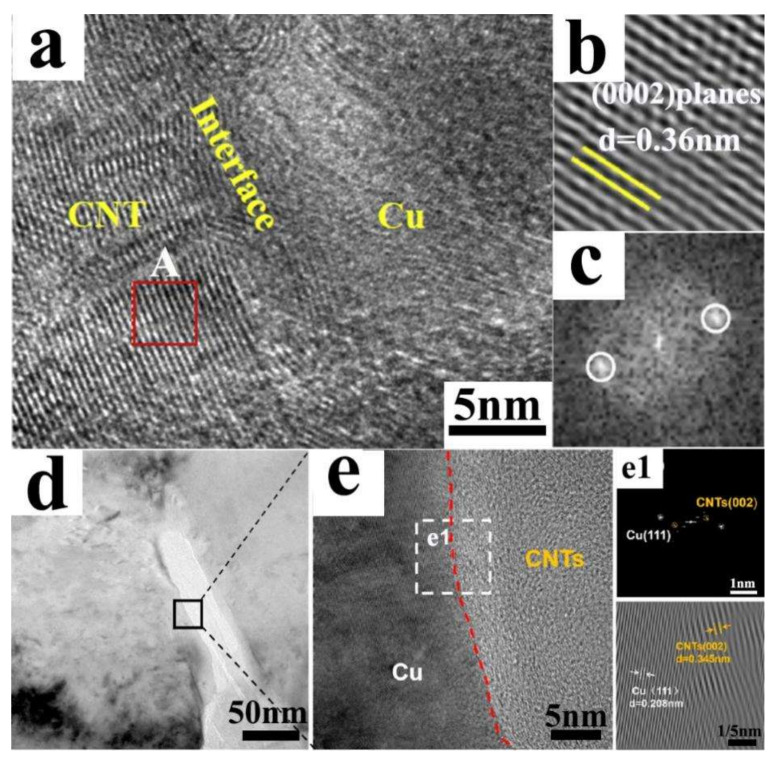
(**a**) HRTEM image of nanostructured CNT/Cu at high magnification, (**b**) IFFT image of CNT embedded in Cu matrix, (**c**) diffraction pattern correspond to part A [67]; (**d**) TEM images of the interface between CNTs and Cu matrix before tensile deformation, (**e**) HRTEM images of the black box in (**d**), (**e1**) FFT patterns and IFFT images of the white box in (**e**) [68].

The physical binding structure predominantly hinges upon van der Waals forces; accordingly, CNT/Cu composites typically manifest inferior properties. Fortuitously, progression in fabrication techniques facilitates the establishment of inter-diffusion structures between Cu and CNTs, thereby intensifying interfacial bond strength, especially subsequent to annealing or high-pressure pressing. Consequently, specimens with physical binding interfaces evince enhanced attributes, particularly with respect to mechanical properties, albeit in specific orientations. Through layer-by-layer brushing of CNTs and Cu sputtering, the Cu/CNT/Cu layered composite attained a Cu-C inter-diffusion structure along with upgraded properties [43]. An alternate approach for augmenting interface bonding encompasses high-pressure pressing; for example, after subjecting to 6 GPa of high-pressure torsion, the interface between CNTs and Cu displays exceptional continuity and a scarcity of physical gaps (Figure 4a–c) [67].

By means of co-deposition, CNTs are not only incorporated within Cu grains or proximate to adjacent Cu grains but also display negligible agglomeration, engendering a tenacious bond with the matrix (Figure 4d,e). This arrangement gives rise to enhanced properties, namely a yield strength of 254 MPa, an ultimate tensile strength of 290 MPa, an elongation of 15%, and a high conductivity of 92.3% IACS [68]. During the co-deposition process, Cu^2+^ ions are reduced to metallic Cu on the CNTs’ surface concurrently while facilitating the reduction of the CNTs themselves; accordingly, the interface is firmly bonded without oxides or detectable voids. Moreover, efficient load transfer constitutes the principal strengthening mechanism.

**Figure 5:**
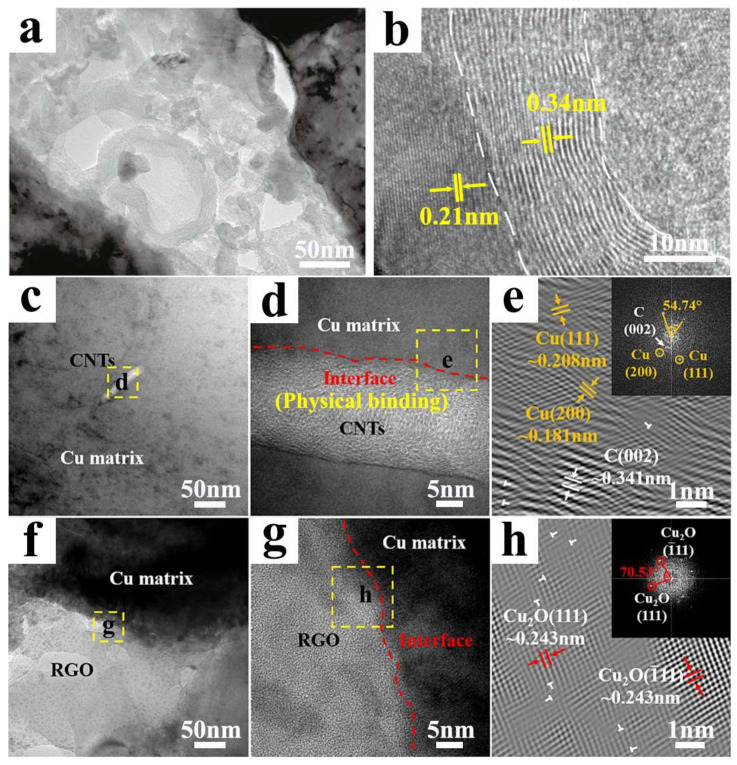
(**a**) TEM microstructure image of the CNT/Cu–Al_2_O_3_ composites, (**b**) the corresponding HRTEM image [65]; (**c**–**e**) TEM images of the 0.064 vol % CNT/Cu_f_^®^Cu composite, (**f**–**h**) TEM images of the 0.064 vol % RGO/Cu_f_^®^Cu composite [69].

With the introduction of Al_2_O_3_ as a catalyst particle formation facilitator and co-reinforcing phase within Cu, CNTs were generated on the Cu-Al_2_O_3_ particles from C_2_H_4_. During this procedure, a robust interfacial bond was established between the CNTs and Cu (Figure 5a,b) [65]. The existence of Al_2_O_3_ enabled the conversion of Cu into a catalyst for C_2_H_4_, leading to a homogeneous distribution of CNTs within the composites. This contributed to the enhancement of mechanical properties while sustaining a high level of electrical conductivity. Similarly, upon the integration of reduced graphene oxide (RGO) to form CuO_2_ within the Cu matrix, CNTs and RGO served as a three-dimensional skeletal reinforcing phase. However, CNTs and Cu merely demonstrated physical binding (Figure 5c–h) [69]. Additionally, CuO_2_ was also detected at the interface between CNTs and RGO, further intensifying the interfacial bond strength within the composite.

The physical binding method represents the epitome of direct and unadulterated means to achieve interface bonding, as it circumvents the introduction of heterogeneous elements at the interface and preserves the intrinsic structure of CNTs. However, owing to the feeble van der Waals force, the bonding strength at the interface remains constrained, thereby resulting in suboptimal overall performance of the composite material.

## 3. Chemical Bonding Interface

Owing to the fragile interfacial interactions between CNTs and Cu, a robust interconnection between carbon (C) and Cu can elicit extraordinary performance in CNT/Cu composites. Specifically, chemically modified CNTs display augmented cohesive strength with the matrix via a chemically bonded coating formed on their surfaces. The adhesion between CNTs and the substrate Cu is markedly augmented, thereby enhancing mechanical properties; for example, the fracture surfaces of CNT/Cu composites differ contingent on the bonding mode at the interface, with chemically modified CNTs demonstrating greater adhesion to Cu compared to their unmodified counterparts [21]. Analogous to the aforementioned layer-by-layer configuration, carboxyl-modified SWCNTs were integrated with Cu in a layer-by-layer manner through electrospinning and physical vapor deposition. The resultant composite manifested superior properties when juxtaposed with pure Cu fabricated using an identical approach [66]. A marginal modification of -COOH groups on SWCNTs (0.01 wt %) concurrently enhanced both the mechanical and tribological properties of Cu [83].

Historically, CNTs are modified with oxygen-containing entities such as carboxyl or hydroxyl groups by utilization of oxidizing agents encompassing H_2_SO_4_, HNO_3_, diluted HNO_3_, and H_2_O_2_ [84]. These oxygen-containing entities facilitate the genesis of Cu_2_O, thereby escalating the wettability between Cu and CNTs and culminating in stronger interfacial adhesion. Nonetheless, excessive functionalization might compromise the structural integrity of CNTs and have an adverse influence on the properties of the composite. Thus, it is indispensable to regulate the functionalization process.

Occasionally, CNTs undergo functionalization with thiol or nitrogen moieties. These functional entities not merely exert an influence on the morphology of deposited Cu but also have a bearing on its chemical state; notably, thiol-modified CNTs evince remarkable property-enhancement effects [14]. Thiol moieties exhibit a conspicuous affinity for specific metals and forge tenacious bonds via sulfur atoms. Moreover, noble metal particles do not agglomerate on the surfaces of thiol-modified CNTs, enabling palladium (Pd) to uniformly nucleate on these surfaces and facilitate the growth of Cu on the CNTs. Owing to the vigorous interaction between thiol-modified CNTs and Cu, the resultant CNT/Cu films demonstrate optimal properties. Additionally, this potent interaction markedly impacts the microstructure of Cu on the surface of CNTs, giving rise to an elongated trapezoidal configuration rather than the spherical forms witnessed in carboxyl- or nitrogen-modified CNTs. Intriguingly, through electroless deposition, nanocrystalline Cu coatings on modified MWCNTs engender uniformly distributed CNTs within the Cu matrix, enhancing interfacial contact and refining the grain structure; consequently, both yield strength and tensile strength escalate to as high as 692 MPa and 865 MPa [46].

Contrariwise, nitrogen-doped and carboxyl-modified CNTs have lone electron pairs that can interact with metals by means of electron pair sharing. Nonetheless, nitrogen-doped CNTs undergo protonation in aqueous media, giving rise to an elevated pH; as a result, these CNTs show the lowest adherence to Cu. The carboxyl modification is generally accompanied by intense oxidation processes that undermine the structural integrity of CNTs, thereby causing a reduction in the conductivity of the resulting CNT/Cu composites [85]. Additionally, prolonged sonication adversely impacts the structural integrity of CNTs [66].

For applications in domains such as catalysis and energy storage, the performance of carboxyl or nitrogen-doped carbon nanotubes loaded with copper is preeminent for nanocomposite materials, as it promotes the dispersion of copper nanoparticles. Moreover, due to their heightened surface energy state and proneness to oxidation, non-conductive oxidized copper can be readily formed, detrimentally affecting conductivity [14]. Subsequently, optimizing the preparation process and structural design can further potentiate performance.

**Figure 6:**
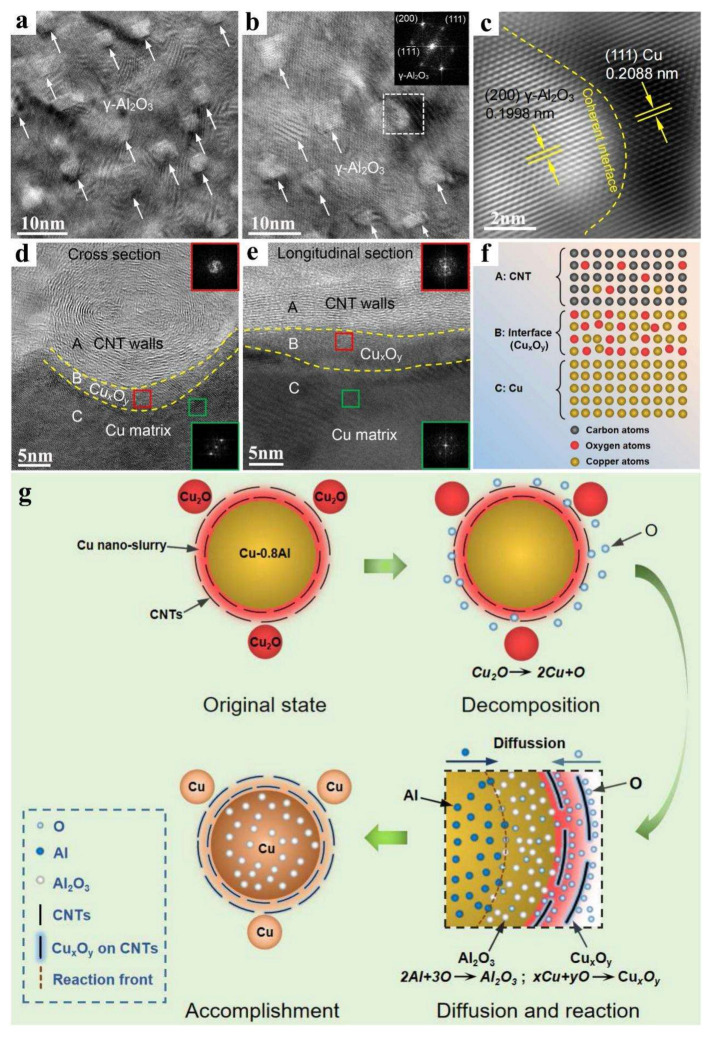
(**a**,**b**) HRTEM image of Al_2_O_3_ distribution together with FFT image (inset of (**b**)), (**c**) coherent interface and interplanar space measurement between Al_2_O_3_ and Cu matrix, (**d**) Cross-sectional image of CNT/Cu interface, (**e**) longitudinal sectional image of CNT/Cu interface, (**f**) schematical illustration of CNT/Cu interface, (**g**) Schematic illustration of oxygen diffusion and in situ solid-reaction for Cu–Al_2_O_3_-CNTs during internal oxidation [77].

For illustration, in contradistinction to the chemical modification schema of CNTs, the internal oxidation of Cu enables the establishment of CNT-Cu_x_O_y_-Cu interfacial bonding, notwithstanding the amorphous nature of the boundary phase (Figure 6) [77]. Comparably, during the brushing procedure, CNTs are oriented under the shear forces imparted by brushing, and the diffusion of Cu into CNTs is facilitated by heat treatment. This can (a) enhance the charge transport between particles by reducing the tunneling barriers or providing conductive shunts around defective non-conductive regions, and (b) exert an influence on the electronic structure of CNTs via charge-transfer doping [43]. The electrospinning process can engender a homogeneous and neatly aligned CNT coating on the surface of copper foil, where thermal treatment expedites the diffusion of Cu into the CNTs, comparable to the copper diffusion witnessed in PVP nitrogen-doped electrospinning solutions. This procedure augments the electron density of the CNT wall, ameliorates its metallic attributes, and optimizes charge-transport properties. Moreover, the diffusion of Cu can give rise to the formation of a percolative conductive network throughout the entire CNT/metal matrix ensemble, which is conducive to attaining high charge capacity (CCC) or enhanced capacitance in carbon-doped Cu composite materials. The escalation in the activation energy for Cu diffusion within these composites has been demonstrated to curb both surface and grain boundary diffusion of Cu, especially at elevated temperatures; this phenomenon exerts a substantive influence on the realization of high CCC or capacitance in Cu/CNT composite materials [66].

**Figure 7:**
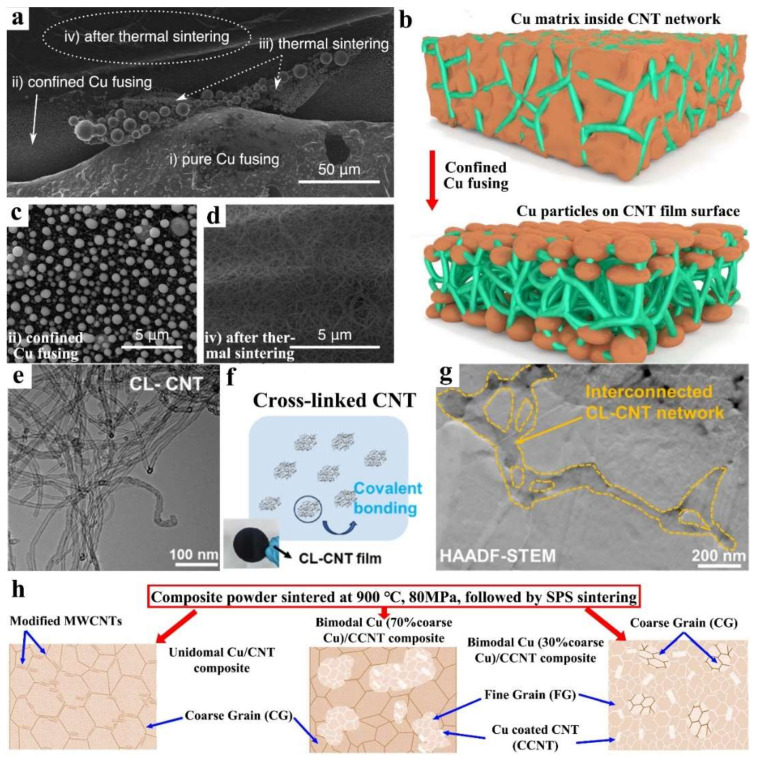
(**a**) SEM image of CNT/Cu composite, including (i) pure Cu fusing of Cu layer, (ii) confined Cu fusing, (iii) thermal sintering of Cu particles, and (iv) empty CNT network after the thermal sintering, (**b**) schematics of the confined Cu fusing, (**c**,**d**) the marked region in (**a**) [70]; (**e**) TEM of CL-CNT, (**f**) dispersion diagram of CL-CNT in the solution during molecular level mixing process, (**g**) TEM of CL-CNT/Cu [72]; (**h**) schematic showing the unimodal and bimodal Cu/MWCNTs composites [73].

In addition to surface modification, the meticulously designed microstructure exerts a profound impact on the properties of composites, such as CNT networks (Figure 7a–d) [70], cross-linked networks of modified CNT-strengthened Cu (Figure 7e–g) [72], or modified CNTs enhancing particle gradation Cu via coarse and fine powder mixing (Figure 7h) [73], which exhibit enhanced attributes. The cross-linked configuration of CNTs retards the growth of surrounding Cu grains and augments the fine-grained strengthening effect. Moreover, load transfer constitutes the predominant strengthening mechanism for both composite systems.

It is widely recognized that chemical bonding exhibits greater durability compared to physical binding. Consequently, CNT/Cu composites with chemically bonded interfaces generally exhibit superior properties in comparison to their physically bonded counterparts (Table 2 and Table 3, Figure 2 and Figure 3). Furthermore, the chemical bonding mode serves as the primary mechanism for interface formation in CNT/Cu composites. Most advancements in interface bonding are derived from this fundamental approach (Table 2 and Table 3). Additionally, the generation of Cu_2_O can enhance the interfacial adhesion between CNTs and the Cu matrix, thereby imparting advantageous effects on the mechanical properties of the composite. However, Cu_2_O may induce electron scattering, which adversely affects the electrical conductivity of the composite [78]. This gives rise to divergent perspectives on electron transport at interfaces formed by introducing oxygen-containing groups into Cu_x_O_y_ structures. Moreover, frequent modification procedures often compromise the integrity of CNTs, leading to deteriorated performance characteristics. Consequently, newly experimental findings have demonstrated that a controlled oxidation modification on the surface of carbon nanotubes not only enhances the interfacial bonding strength between CNT and Cu but also facilitates interfacial electron transport [86].

## 4. Metallurgical Bonding Interface

The incorporation of metals to form a metallurgical bonding interface with Cu instead of physical binding or chemical bonding can augment the wettability of CNTs and Cu, constituting another efficacious approach for attaining a high-adhesion interface and thereby circumventing the aforementioned disadvantages. Ag and Cr are prevalently utilized to forge metallurgical bonding interfaces, as both are capable of forming solid solutions with Cu and effectively moistening the surface of CNTs.

**Figure 8:**
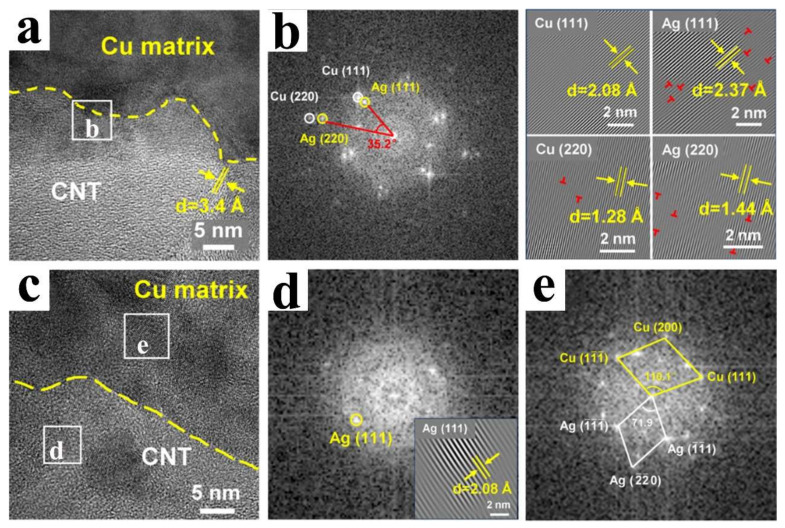
HRTEM images with corresponding FFT and IFFT images of interface areas in 0.75 CNT-Ag/Cu composite, (**b**) is IFFT image of the area marked in (**a**,**d**,**e**) are IFFT images of the areas marked in (**c**) [78].

Conventionally, Ag, Ni, or Cr nanoparticles are electrochemically deposited on the surface of CNTs, subsequently, the nanocomposites are incorporated into the Cu matrix by means of ball milling and spark plasma sintering (SPS). The nucleation and insertion of Ag nanoparticles at the defects of CNTs can boost both the graphitization degree and electrical conductivity of CNTs (Figure 8) [78]. Additionally, the ambiguous interface between CNTs and Cu implies that Ag nanoparticles facilitate the mutual diffusion of Cu, Ag, and C atoms during the sintering process.

Ni functions not merely as a catalyst in the arc discharge synthesis of CNTs but also augments the interface wettability between CNTs and Cu by enabling metallurgical bonding. The outcomes of molecular dynamics simulations disclose that the interfacial bonding between CNTs and Cu can be enhanced, and the interface vacancies mitigated, upon the introduction of Ni atoms [87]. Reaping the benefits of Ni nanoparticles on the surface of SWCNTs, the fracture mode undergoes a transformation from intergranular in pure Cu to ductile in the composite [79].

The introduction of metallic elements to establish a metallurgical bond at the interface between CNTs and the Cu matrix represents a promising approach, capable of significantly enhancing the bonding strength at the interface and further improving the mechanical properties of composite materials. However, the impact of metallurgically bonded interfaces on the electrical conductivity of composites remains unclear (Figure 2 and Figure 3), as it is influenced by factors such as distribution of alloying elements on CNT surfaces, coverage degree and uniformity of metallurgical bonded interfaces, and electron-transfer mechanism across interfaces. Additionally, the limited availability of suitable metals for interface formation and the lack of well-defined interactions with CNT surfaces have resulted in a scarcity of relevant studies. As a result, metallurgical bonding is predominantly combined with physical or chemical bonding methods to enhance interface strength and establish composite interfaces.

## 5. Multiple Interface Bonding Modes

**Figure 9:**
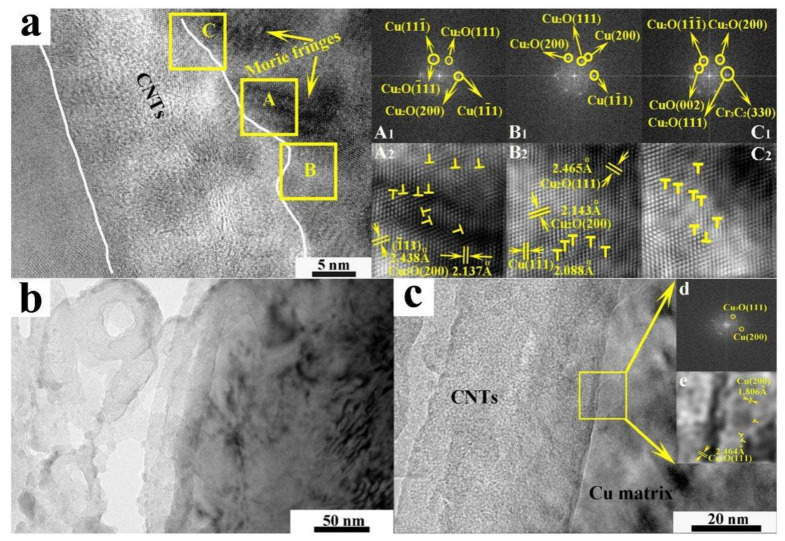
Interface of (**a**) Cu/CNTs (alloying method prepared CuCr alloy) and (**b**) Cu/CNTs-1 (co-deposition method prepared CuCr alloy) composites (FFT (**d**) and IFFT (**e**) of the marked box). A_1_ (A_2_), B_1_ (B_2_), and C_1_ (C_2_) are FFT (IFFT) images of the region A, B, and C, (**c**) interface of Cu/CNTs-1, (**d**) and (**e**) are FFT and IFFT images of box in (**c**) [64].

**Figure 10:**
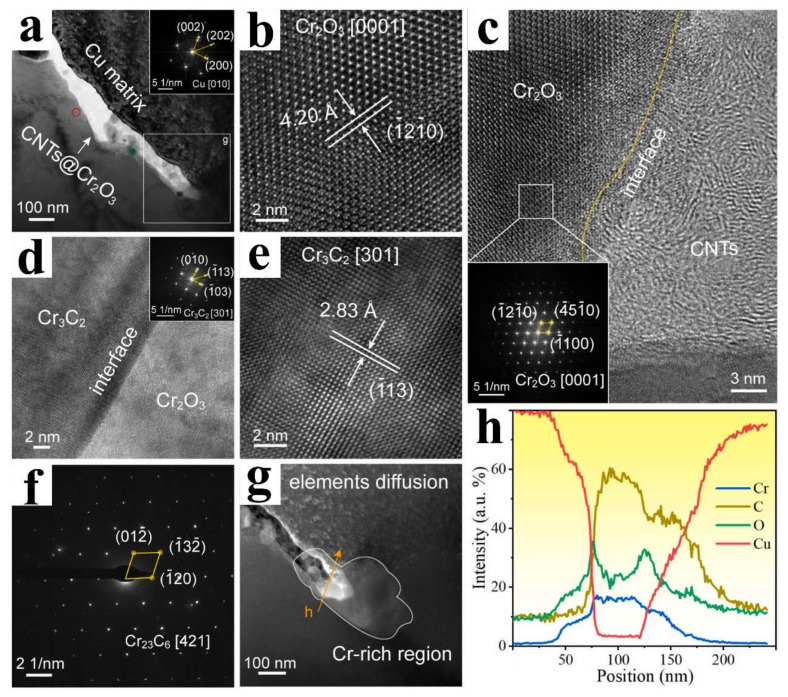
(**a**) BF-TEM image, the inserted SAED pattern derives from the red circle area in (**a**), (**b**) HRTEM images of the in situ formed Cr_2_O_3_, (**c**) HRTEM image of the interfacial structure between Cr_2_O_3_ and CNTs, the inserted FFT image derives from the white square area marked in (**c**), (**d**) HRTEM image of the interfacial structure between Cr_3_C_2_ and Cr_2_O_3_, the inserted FFT image derives from the Cr_3_C_2_ in (**d**), (**e**) HRTEM images of the Cr_3_C_2_ in (**d**), (**f**) SAED pattern of the Cr_23_C_6_ record from the green circle area in (**a**), (**g**) the magnified DFTEM image in the white square marked g in (**a**), (**h**) elemental line scanning results along the marked orange line in (**g**) [80].

Moreover, diverse interfacial bonding modalities between CNTs and Cu have been established, manifesting enhanced attributes. Employing a CuCr alloy as the catalyst and generating Cr_3_C_2_ and Cu_2_O to enhance the interfacial wettability of CNTs with the Cu matrix, CNT/Cu composites were fabricated via the chemical vapor deposition (CVD) growth of CNTs and subsequent SPS (Figure 9) [64]. In contrast to pure Cu, the electrical conductivity of CNT/Cu is lower on account of the incorporation of CNTs; precisely, the hollow-core CNTs hinder electron transportation within Cu. On the contrary, the hardness and tensile strength of CNT/Cu are 24.7% and 36.1% higher, respectively, compared to those of pure Cu. Simultaneously, thanks to the stronger bonding between CNTs and Cu and the more homogeneous dispersion of CNTs in the matrix, superior properties are witnessed in the CNT/Cu composites. Through controlled oxidation using H_2_SO_4_ and subsequent unzipping along with the formation of CrC_x_ (including Cr_3_C_2_, Cr_7_C_3_, or Cr_23_C_6_), modified CNTs augment the physical contact area with Cu and improve the interfacial wettability (Figure 10) [80,81].

The carbides formed at the interface between the CNTs and the Cu matrix not only elevate the wettability but also enhance the mechanical attributes of the composites by means of an interfacial pinning effect. Comparably, the introduction of Sn and Ag as sensitizing entities facilitates the generation of Cu_2_O on the surfaces of CNTs, thereby intensifying the interfacial bonding strength through the diffusion of Cu_2_O and atoms (Sn and Ag) into the Cu matrix, where chemical bonding reigns as the principal mode of adhesion [82]. Moreover, with the aid of TiC, the composite further augments yield strength and ultimate tensile strength by 13.4% and 5.8%, respectively [88].

The performance of composite materials can be further enhanced in specific aspects by leveraging the strong bonding strengths exhibited by two existing interfaces (Figure 2 and Figure 3). However, a more comprehensive mechanism study is required for composites featuring multiple interface bonding due to the current lack of mature analysis in metallurgical bonding research.

## 6. Mutual Infiltration Interface

Chemical bonding can undermine the integrity of carbon nanotubes. While the introduction of copper oxide can fortify the interfacial bonding between the two, thereby facilitating the improvement of mechanical properties, it concurrently elicits a certain extent of electron scattering, thereby reducing conductivity. Metallurgical bonding can circumvent these predicaments; nonetheless, it demands complete and homogeneous coating of carbon nanotubes, which presents low controllability. Once there exists uncoated carbon nanotubes, defects will arise, adversely influencing the performance of the composite material. Since the introduction of the reinforcing phase is restricted, the improvement of performance will also be constrained.

Owing to the inherent propensity for agglomeration of CNTs, the performance typically undergoes an initial escalation followed by a descent as the quantity increases. Generally, the quantity is rather low. After reaching the critical value, the further augmentation of the amount of the reinforcing phase will give rise to a decrease in the base performance due to the transitional agglomeration of the reinforcing phase or the emergence of poor interfacial wetting. Consequently, this leads to an increase in the brittleness of the composite material and ultimately results in a deterioration of its mechanical properties [78]. Graphene oxide (GO) and acid-treated CNT hybrid reinforcement in a Cu matrix can form an interconnected network and exhibited synergistic strengthening effects, and resulted in composites with hardness of 226.8 HV_0.1_, thermal conductivity of 527 W/m·K and friction coefficient of 0.62 [89].

The occurrence of high-density interfacial dislocations and interfacial disordered domains between the copper matrix and CNTs gives rise to the establishment of robust interfacial adhesion. The impregnation of copper into the tube walls not only alleviates electron transport impediments but also augments the interfacial shear strength between the Cu matrix and CNTs. The incorporation of copper within CNTs optimizes the point-to-point cross-sectional area of CNTs, thereby minimizing the resistivity within the tubes. Moreover, Cu infiltration and surface coating effectively alleviate the agglomeration of CNTs and enhance their dispersion within the matrix. Therefore, we propose utilizing the unique structure of aCNTs to fabricate a nanoscale mesh intercalated composite interface in order to enhance the bonding strength between the reinforced phase and the matrix. Firstly, aCNTs were dispersed in a copper brine solution (e.g., copper nitrate or copper sulfate) for complete contact mixing, followed by employing a hydrogen reduction process to obtain Cu and aCNT nanocomposite powder. Subsequently, this powder is mixed with copper powder and subjected to SPS to produce a cylindrical composite material with an electrical conductivity of 82.5% IACS and a nano-hardness of 1387 MPa. Furthermore, successful synthesis of a composite nanopowder containing Cu within both the pores and mesoporous wall of aCNTs has been achieved, which are denoted as Cu in the mesoporous side wall or pores of aCNTs.

## 7. Conclusions and Prospect

Based on the extensive application scenarios of copper-based composites and the tremendous potential of carbon-nanotube-reinforced metal-based composites, abundant research achievements have been accumulated for carbon-nanotube-reinforced copper-based composites. In the current studies, the strengthening mechanism of CNTs has also been investigated thoroughly. In composites, the interface exerts a significant influence on performance. Therefore, in view of the interface between CNTs and Cu, this work summarized different interfaces of CNT/Cu composites, which are mainly physical bonding, chemical bonding, and metallurgical bonding, or a mixture of different interface bonding approaches, while chemical bonding is the most studied and most foundational one. The formation of cupric oxide through chemical bonding enhances the interfacial wettability between CNT and Cu, thereby facilitating metallurgical bonding and composite interfaces, ultimately leading to improved performance of CNT/Cu. Nevertheless, the majority of them are concentrated on the macroscopic optimization of the interface between CNTs and the copper matrix, making it difficult to achieve more refined interface regulation. Additionally, due to the inherent characteristics of CNTs and Cu, it is challenging to further enhance the interface bonding strength between them, which has led to a bottleneck in the research of CNT/Cu composites.

Therefore, to enhance electron transport and load transfer at the micro-interface of CNT/Cu composites and further improve their overall performance, future research should address several aspects: (a) Utilizing advanced computer technology to strengthen theoretical investigations and employing numerical simulation and machine learning methods for accurate identification of micro-interface structures that yield superior comprehensive properties for CNT/Cu composites; (b) Enhancing experimental techniques and methodologies to precisely control the interface of CNT/Cu composites; (c) Optimizing post-treatment technologies for refining the interface structure of CNT/Cu composites accurately and enhancing composite properties; (d) The valence variation of Cu during the excavation and regulation of CNT/Cu preparation and its impact on the overall properties of composites; (e) Advancing large-scale preparation techniques for enabling true industrial applications of CNT/Cu composites. Building upon these advancements, increasing the proportion of CNTs will achieve genuine lightweight characteristics in CNT/Cu composites, ultimately enhancing energy efficiency of components. Once the actual performance of CNT/Cu composites reaches theoretical levels in the future, they are expected to fully replace existing copper matrix composites, particularly in light-conducting applications with great potential impact on power systems in automobiles, ships, aircraft, etc.

## Data Availability

No new data were created.
